# Movable Seat Dentist's Chair

**Published:** 1853-01

**Authors:** 


*Movable Seat Dentist's Chair.*—Mr. A. M. Asay, Dentist, of Philadelphia, has, within the last few years, invented a chair for dentists, which combines more advantages than any which we have seen. For a description of it, the reader is referred to his advertisement on the cover of the Journal.

